# Dense Hydrogen-Bonded Assembly of Hydrogen-Rich Cations and Pentazolate Anions: A Series of Highly Insensitive Ionic Salts

**DOI:** 10.3390/molecules30122613

**Published:** 2025-06-16

**Authors:** Lianghe Sun, Hongwei Zhu, Shuaijie Jiang, Xiaofeng Yuan, Guoping Lu, Ming Lu, Yuangang Xu

**Affiliations:** School of Chemistry and Chemical Engineering, Nanjing University of Science and Technology, Nanjing 210094, China; 18112254480@163.com (L.S.); 18895545396@163.com (H.Z.); jsj2020@njust.edu.cn (S.J.); yuan_xiaof@163.com (X.Y.); glu@njust.edu.cn (G.L.)

**Keywords:** pentazolate anion, hydrogen bond, crystal structures, mechanical sensitivity

## Abstract

Compounds containing the pentazolate anion (*cyclo*-N_5_^−^) represent a distinctive group of energetic materials that have received extensive attention in recent years. *Cyclo*-N_5_^−^ was used as a polynitrogen anion for the syntheses of energetic salts through metathesis reactions. Propamidinium (**1**), 5-amino-4-carbamoyl-1*H*-imidazol-3-ium (**2**), (1*H*-1,2,3-triazol-4-yl)methanaminium (**3**), 5-amino-4*H*-1,2,4-triazol-1-ium (**4**), 5-amino-3-methyl-4*H*-1,2,4-triazol-1-ium (**5**), and amino(pyrimidin-2-yl)methaniminium (**6**) pentazolates were obtained with high yields (>80%), and their crystal structures were confirmed through single-crystal X-ray diffraction analyses. Hirshfeld surface analyses and 2D fingerprint plots generated by CrystalExplorer17 demonstrated that these compounds exhibited extensive hydrogen-bonding networks in their crystal packing. Mechanical sensitivity tests showed that all the prepared salts were highly insensitive (IS > 35 J, FS > 360 N), providing valuable insights for the further exploration of broader energetic materials containing *cyclo*-N_5_^−^.

## 1. Introduction

Nitrogen-rich energetic materials represent a significant category of high-energy density materials that have significant applications in energetic systems, including propellant formulations, explosive compositions, and advanced energy storage technologies [1,2,3,4,5]. These nitrogen-rich compounds exhibited distinctive molecular architectures featuring a high density of N−N, C=N, and C−N bonds, which collectively enhance their positive heats of formation. The remarkable energy output characteristics of these materials are primarily derived from their substantial heats of formation, enabling the exothermic production of highly stable nitrogen gas (N_2_) upon molecular decomposition. This characteristics position them as a highly promising category of eco-friendly energetic materials [6,7,8]. Building upon nitrogen-rich energetic materials, new investigations have revealed that converting them into ionic nitrogen-rich energetic salts can further enhance their performance. Compared to their neutral counterparts and conventional molecular energetic materials, nitrogen-rich energetic salts typically demonstrate superior physicochemical properties including reduced volatility, increased density, and enhanced thermal stability [9,10,11]. Among various nitrogen-rich structural motifs (such as pyrazole, tetrazole, and triazole derivatives), *cyclo*-pentazolate (*cyclo*-N_5_^−^) salts exhibit remarkable features, most notably their highly positive enthalpy of formation and environmentally friendly nitrogen gas being the primary detonation product. The molecular structure of *cyclo*-N_5_^−^ salts contains metastable N-N and N=N bonds, which can release substantial dissociation energy upon conversion to the highly stable N≡N triple bonds. This distinctive property endows *cyclo*-N_5_^−^ salts with broad application prospects in high-energy material fields, including propulsion systems, explosive charges, and pyrotechnic compositions [12,13].

However, in the development of *cyclo*-N_5_^−^ salts, structural stabilization has always been the core challenge in design and experimental synthesis, as their stability directly determines the material’s storage performance and application value. Although the *cyclo*-N_5_^−^ anion possesses extremely high energy density, its metastable nature poses significant challenges for storage and practical applications [14,15]. Non-metallic cation-based *cyclo*-N_5_^−^ salts may achieve kinetic stabilization through hydrogen bonding networks. In contrast with the typical neutral hydrogen bonds found in conventional CHON-based energetic materials (carbon (C), hydrogen (H), oxygen (O), and nitrogen (N)-based energetic compounds, exemplified by TNT, RDX, HMX, and CL-20), these salts form unique charged hydrogen bond systems despite the absence of covalent interactions between ions. These hydrogen bonds exist not only within intramolecular structural units but also extensively in intermolecular interactions, ultimately constructing complex multidimensional hydrogen bonding networks. Both theoretical and experimental studies have confirmed that hydrogen bonds play a crucial stabilizing role in ionic systems containing *cyclo*-N_5_^−^ salt, endowing these materials with the advantage of low sensitivity [16,17].

To develop non-metallic *cyclo*-N_5_^−^ salts with low sensitivities, we designed a novel series of compounds by utilizing the hydrogen-bond networks in ionic systems. We hypothesized that introducing a dense hydrogen-bond network would reduce the sensitivity of these *cyclo*-N_5_^−^ salts while preserving their energetic performance. Both theoretical calculations and experimental characterizations consistently confirmed their low sensitivities and acceptable energetic properties, thereby validating our design strategy.

## 2. Results and Discussion

### 2.1. Design and Synthesis

**Scheme 1:**
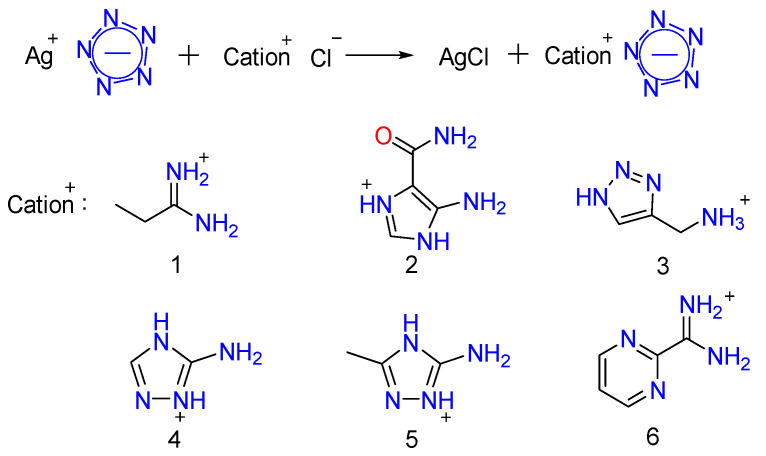
Syntheses of *cyclo*-N_5_^−^ salts **1**–**6**.

Using a method developed by our group, silver pentazolate (AgN_5_) was successfully synthesized [18]. All target salts were successfully synthesized by the metathesis reactions of AgN_5_ with the corresponding hydrochloride salts [19], and all the yields exceeded 80%. Compounds **1**–**6** were synthesized as shown in Scheme 1. It was found that salts **1**–**6** were easily soluble in CH_3_CH_2_OH, CH_3_OH, and H_2_O. After evaporation at room temperature for 3–5 days, crystals were formed, and their molecular structures were uniquely determined by single-crystal X-ray diffraction.

### 2.2. Crystal Structures

**Figure 1:**
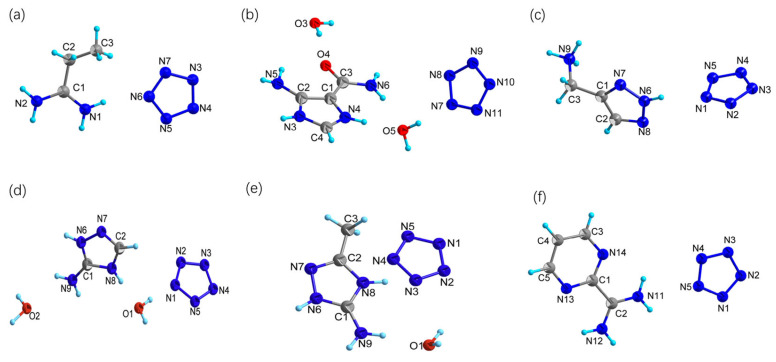
(**a**–**f**) Thermal ellipsoid diagrams (50% probability level) of compounds **1**, **2**∙2H_2_O, **3**, **4**∙2H_2_O, **5**∙H_2_O, and **6**.

To gain deeper insights into the structural characteristics and intermolecular interactions of compounds **1**–**6**, single-crystal X-ray diffraction analyses were performed. Single crystals of compounds **2**, **4**, and **5** were obtained by slow evaporation from aqueous solution, while compounds **1**, **3**, and **6** were crystallized from methanol. The molecular structures of these compounds are presented in Figure 1.

**Figure 2:**
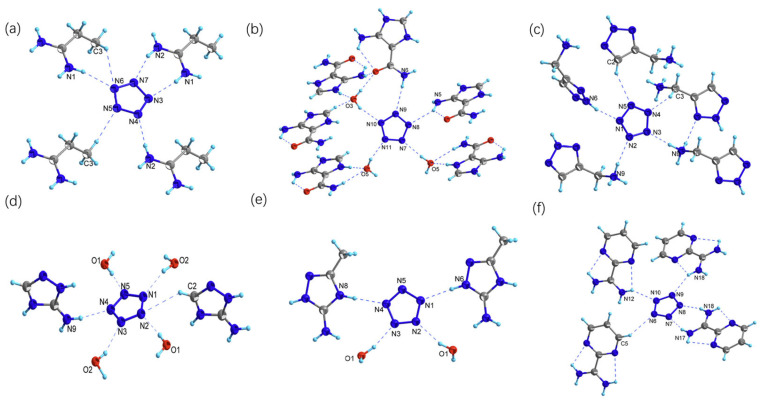
(**a**–**f**) Hydrogen bonds of the *cyclo*-N_5_^−^ for compounds **1**, **2**∙2H_2_O, **3**, **4**∙2H_2_O, **5**∙H_2_O, and **6**. Dashed lines indicate strong hydrogen bonding.

**Figure 3:**
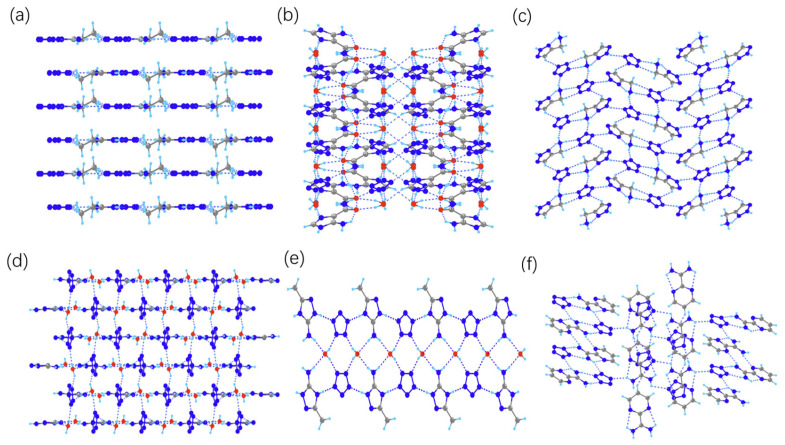
(**a**–**f**) The 3D structural layer network for compounds **1**, **2**∙2H_2_O, **3**, **4**∙2H_2_O, **5**∙H_2_O, and **6**. Dashed lines indicate strong hydrogen bonding.

Compound **1** crystallized in the monoclinic space group *P*2_1_/*m*, with two molecules in the lattice units (*Z* = 2). As shown in Figure 2a, the asymmetric unit crystallography consists of one propamidinium cation and one *cyclo*-N_5_^−^ anion. As shown in Figure 2a, each *cyclo*-N_5_^−^ anion achieved stabilization via six distinct hydrogen bonds donated by four propamidinium cations (N1−H1A∙∙∙N3 = 2.20 Å; N1−H1B∙∙∙N6 = 2.13 Å; N2−H2A∙∙∙N7 = 2.15 Å; N2−H2B∙∙∙N4 = 2.14 Å; C3−H3B∙∙∙N5 = 2.55 Å; C3−H3C∙∙∙N6 = 2.61 Å). The N−N bond lengths in *cyclo*-N_5_^−^ are 1.311(3), 1.319(3), 1.320(3), 1.316(3), and 1.320(3) Å, with an average N−N bond distance of 1.3172 Å. Compound **1** features layer-by-layer stacking (Figure 3a).

Compound **2**∙2H_2_O crystallized in the monoclinic space group *C*2/*c*, with eight molecules in the lattice units (*Z* = 8). As shown in Figure 2b, the asymmetric unit crystallography consists of one 5-amino-4-carbamoyl-1*H*-imidazol-3-ium cation, one *cyclo*-N_5_^−^ anion, and two water molecules. The average N−N bond distance in the *cyclo*-N_5_^−^ anion is 1.318 Å. As shown in Figure 2b, each *cyclo*-N_5_^−^ anion achieved stabilization via five hydrogen bonds from two 5-amino-4-carbamoyl-1*H*-imidazol-3-ium cations (N8−H5A∙∙∙N5 = 2.29(2) Å; N9−H6A∙∙∙N6 = 2.22(2) Å) and three water molecules: (O3−H3A∙∙∙N10 = 2.05(2) Å; O5−H5C∙∙∙N7 = 1.99(2) Å; O5−H5D∙∙∙N11 = 2.0.3(2) Å). The lengths of the hydrogen bonds are shorter than the sum of the van der Waals (vdW) radii (rw(N) + rw(N) = 3.20Å); therefore, a dense hydrogen bond network formed.

Both **3** and **4**∙2H_2_O crystallized in the monoclinic space group *P*2_1_/*c*, with four molecules in the lattice units (*Z* = 4) (Figure 2). The average N–N bond distances in **3** and **4**∙2H_2_O are 1.315 Å and 1.3108 Å, respectively, which are slightly shorter than that in **2**. Each nitrogen atom of *cyclo*-N_5_^−^ in **3** and **4**∙2H_2_O serves as a hydrogen bond acceptor (Figure 3c,d); this results in the formation of dense 3D hydrogen-bonding networks.

Compound **5**∙H_2_O is a crystal belonging to the *Fdd*2 space group of the orthorhombic system, with sixteen molecules in the lattice units (*Z* = 16). The asymmetric unit crystallography consists of one 5-amino-3-methyl-4*H*-1,2,4-triazol-1-ium cation, one *cyclo*-N_5_^−^ anion, and one water molecule (Figure 2e). The average N−N bond distance in the *cyclo*-N_5_^−^ anion is 1.3178 Å. Each *cyclo*-N_5_^−^ anion achieved stabilization via four hydrogen bonds from two 5-amino-3-methyl-4*H*-1,2,4-triazol-1-ium cations (N6−H6∙∙∙N1 = 2.03(5) Å; N8−H8∙∙∙N4 = 1.98(5) Å) and two water molecules (O1−H1A∙∙∙N2 = 2.01(5) Å; O1−H1B∙∙∙N3 = 1.93(7) Å). The N and C atoms of the 5-amino-3-methyl-4*H*-1,2,4-triazol-1-ium cations act only as hydrogen bond donors (Figure 3e). Unlike other compounds, in compound **5**∙H_2_O, only four nitrogen atoms in each *cyclo*-N_5_^−^ unit act as hydrogen bond acceptors (Figure 3e).

Compound **6** crystallized in the triclinic space group *P*-1, with four molecules in the lattice units (Z = 4). As shown in Figure 2f, the asymmetric unit crystallography consists of one 2-amino(pyrimidin-2-yl)methaniminium cation and one *cyclo*-N_5_^−^ anion. As shown in Figure 2f, each *cyclo*-N_5_^−^ anion is stabilized through five hydrogen bonds formed by four amino(pyrimidin-2-yl)methaniminium cations and its five nitrogen atoms (C5−H5∙∙∙N6 = 2.59 Å; N17−H17A∙∙∙N7 = 2.07 Å; N18−H18A∙∙∙N8 = 2.06 Å; N18−H18B∙∙∙N9 = 2.11 Å; N12−H12B∙∙∙N10 = 2.29 Å). The five nitrogen atoms of *cyclo*-N_5_^−^ in **6** are not perfectly coplanar, as evidenced by the largest torsion angle of 0.9° (N4−N3−N2−N1). The average N−N bond distance in the *cyclo*-N_5_^−^ anion is 1.3146 Å.

In compounds **1**, **2**∙2H_2_O, **3**, **4**∙2H_2_O, **5**∙H_2_O, and **6**, the average N–N bond lengths in *cyclo*-N_5_^−^ are 1.3172, 1.318, 1.315, 1.3108, 1.3178, and 1.3146 Å. The average N–N bond distance is shorter than that of most reported *cyclo*-N_5_^−^ salts, indicating aromatic characteristics, which are consistent with stable *cyclo*-N_5_^−^ salts [20,21,22,23]. Experimental data reveal that the hydrogen bond distances in this system range from 1.93 to 2.61 Å (Tables S6, S10, S14, S18, S22 and S26), which are significantly shorter than those in conventional *cyclo*-N_5_^−^ salts [19], suggesting stronger hydrogen-bonding interactions. Salts **1**, **2**∙2H_2_O, **3**, **4**∙2H_2_O, **5**∙H_2_O, and **6** exhibit dense hydrogen-bonding interactions between the *cyclo*-N_5_^−^ anions and adjacent cations, as well as water molecules, as clearly shown in Figure 3. This result is further supported by Hirshfeld surface analysis, where a distinct spike is observed in the lower-left corner of the 2D fingerprint plot (N⋅⋅⋅H interactions > 77%)

### 2.3. Vibrational Spectroscopy

**Figure 4:**
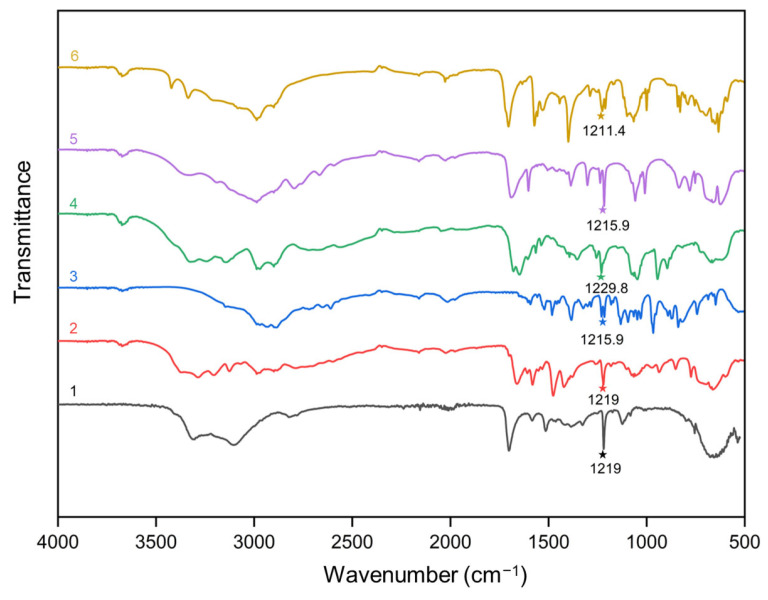
IR spectra of compounds **1**–**6**.

The infrared (IR) spectra of compounds **1**–**6** were identified as providing valuable insights into intramolecular and intermolecular vibrational bonds. From the IR spectra of the six salts (Figure 4), it can be observed that the infrared vibrational absorption of *cyclo*-N_5_^−^ lies between 1211 and 1229 cm⁻^1^, which shows excellent agreement with the frequencies reported for *cyclo*-N_5_^−^ salts (NH_4_N_5_, N_2_H_5_N_5_, and [Na(H_2_O)(N_5_)]⋅2H_2_O) [19,20]. The stretching vibrations of the N–H bonds in **1**–**6** were observed between 3500 and 3000 cm⁻^1^.

### 2.4. Thermal Stability

**Figure 5:**
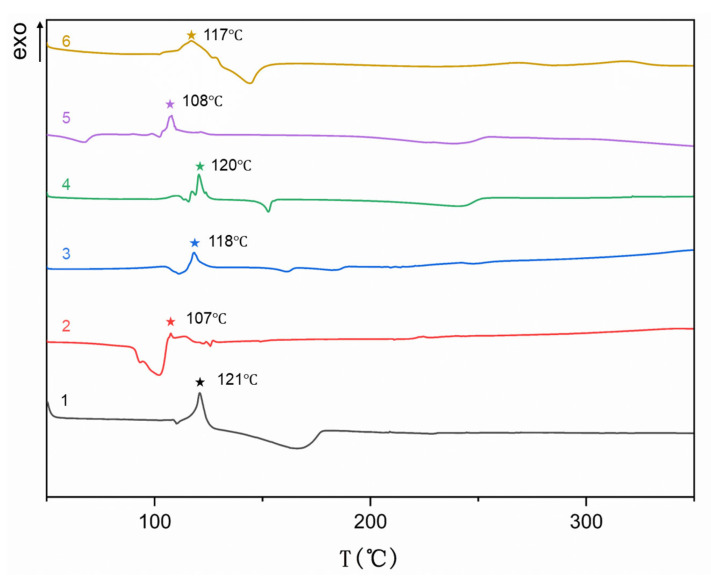
DSC curves for **1**–**6** at a heating rate of 5 °C min^−1^.

The thermal stabilities of compounds **1**–**6** were investigated using differential scanning calorimetry (DSC). As shown in Figure 5, compounds **1**–**6** all decomposed (**1**:121 °C; **2**:107 °C; **3**:118 °C; **4**:120 °C; **5**:108 °C; **6**:117 °C) without melting in the range of 50–300 °C. The thermal analyses in this study demonstrate that dense hydrogen-bonding networks enhance the thermal stabilities of *cyclo*-N_5_^−^ salts. Salts **1**–**6** exhibit higher decomposition temperatures (*T*_peak_ ≥ 107 °C) compared to N(CH_3_)_4_N_5_, [DAG]N_5_, and [EDA](N_5_)_2_ [14,19,22].

### 2.5. Weak Interactions

To understand the interactions involving *cyclo*-N_5_^−^, the CrystalExplorer17 [24] program was employed to generate and analyze the two-dimensional (2D) fingerprint plots and Hirshfeld surfaces of the crystals. These results were utilized to assess the weak interactions in *cyclo*-N_5_^−^ salts, where the red and blue regions correspond to high and low close-contact densities, respectively [25].

**Figure 6:**
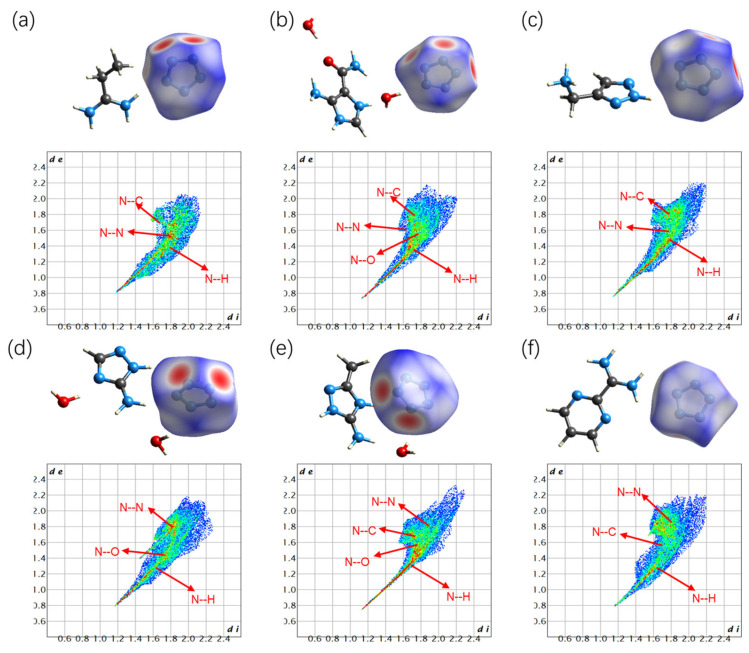
(**a**–**f**) Hirshfeld surfaces and 2D fingerprint plots of *cyclo*-N_5_^−^ in compounds **1**, **2**∙2H_2_O, **3**, **4**∙2H_2_O, **5**∙H_2_O, and **6**.

**Figure 7:**
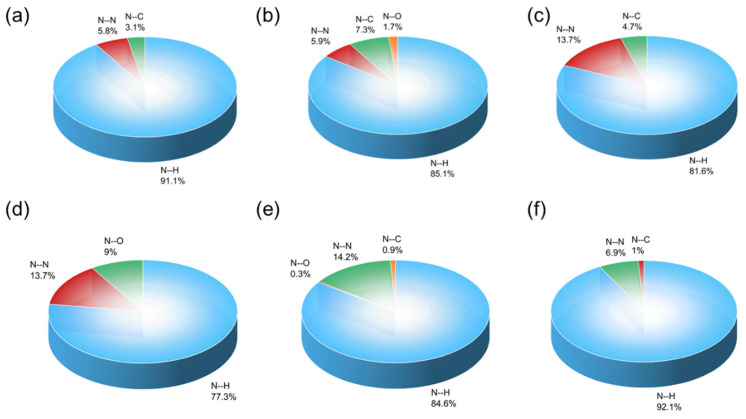
(**a**–**f**) Individual atomic contact percentage contributions of *cyclo*-N_5_^−^ in compounds **1**, **2**∙2H_2_O, **3**, **4**∙2H_2_O, **5**∙H_2_O, and **6**.

For **1**, **2**∙2H_2_O, **3**, **4**∙2H_2_O, **5**∙H_2_O, and **6**, a prominent peak at the bottom left (N⋅⋅⋅H interactions) can be seen in the 2D fingerprint plots (Figure 6). These N⋅⋅⋅H contacts contribute more than 77.3% of the total interactions (Figure 7), indicating that hydrogen bonding plays a dominant role in driving the formation of *cyclo*-N_5_^−^-based crystals. The significant contribution of hydrogen bonds not only stabilizes the crystal structure but also facilitates the arrangement of *cyclo*-N_5_^−^ anions and cations into a well-defined layered or 3D network. This highlights the critical importance of hydrogen bonding in the design and synthesis of *cyclo*-N_5_^−^-based energetic materials with enhanced stability and performance.

### 2.6. Physical Chemistry and Energetic Properties

The solid-phase heats of formation for anhydrous **1**–**6** were calculated using the gas phase heats of formation and heats of phase transition (lattice energy) based on Hess’s law of constant summation (Born–Haber energy cycle; see the Supplementary Material) [19,26,27]. The heats of formation of the *cyclo*-N_5_^−^ anion and the corresponding cations were calculated using the Gaussian 09 (Revision A.02) suite of programs [28,29,30]. Salts **3** and **4** exhibit relatively high heats of formation, reaching 533.86 kJ mol⁻^1^ and 440.9 kJ mol⁻^1^, respectively (Table 1).

The detonation properties of compounds **1**–**6** were theoretically estimated based on experimental densities and calculated heats of formation using the EXPLO5 program (version 6.05.04) [31]. The calculated detonation velocity (*D*) and pressure (*P*) values fall in the range of 7122–8288 m s^−1^ and 14.6–25.1 GPa (Table 1). The detonation velocities of compounds **3** and **4** are higher than that of 3,4-diamino-1,2,4-triazolium pentazolate, N(CH_3_)_4_N_5_, and NH_4_N_5_ [14,19].

### 2.7. Mechanical Sensitivity

The sensitivity is a key indicator for evaluating the safety and practical usability of energetic materials. To analyze the sensitivity properties of anhydrous **1**–**6**, both the impact sensitivity (IS) and friction sensitivity (FS) were measured using the standardized BAM test procedures [32]. Remarkably, all synthesized salts demonstrated exceptionally low sensitivities, with impact sensitivity values exceeding 35 J and friction sensitivity values surpassing 360 N (Table 1). These results demonstrate that the materials are highly resistant to inadvertent initiation, rendering them suitable for operational and long-term storage under practical conditions.

Low sensitivities can be attributed to the synergistic effect of a highly ordered crystalline packing structure and intermolecular hydrogen bonding interactions. Specifically, the 3D network structure constructed through dense hydrogen bonds can significantly enhance the lattice energy of the crystal, thereby greatly improving its structural stability. As shown in Table 1, salts **1**–**6** exhibit lower mechanical sensitivities than N(CH_3_)_4_N_5_, [DAG]N_5_, and [EDA](N_5_)_2_ [14,19,22]. Such properties are highly desirable for the development of next-generation energetic materials with enhanced safety profiles.

## 3. Materials and Methods

During experimental operations, extra care must be taken when handling these high-energy compounds, and masks, gloves, and goggles must be worn throughout the entire process. The necessary protective equipment must be provided and close at hand.

### 3.1. Reagents and Instruments

All chemicals employed in this study were analytical-grade materials procured from domestic manufacturers in China and used without further purification. ^1^H NMR (500 MHz) and ^13^C NMR (125.72 MHz) spectra were recorded using a Bruker AVANCE III 500 spectrometer (Germany). Differential Scanning Calorimetry (DSC) studies were carried out using a NETZSCH DSC 204 F1 Phoenix instrument (Germany) operated in a nitrogen atmosphere with a heating rate of 5 °C min^−1^. IR spectra were performed with a Bruker ALPHA II Fourier transform infrared spectrometer (Germany). The single-crystal X-ray diffraction measurements for **1**–**6** were conducted using a Bruker Smart Apex II diffractometer (Germany) using Mo-Kα radiation (*λ* = 0.71073 Å) or Cu-Kα radiation (*λ* = 1.54178 Å) with a graphite monochromator at temperatures of 150, 170, 173, and 296 K. An Anton Paar Ultrapyc 5000 gas pyrometer (Austria) operating at 25 °C was employed to determine the densities of the samples. Impact sensitivity was tested with a BAM Fall-hammer (Czech), while friction sensitivity was assessed using a BAM friction tester (Czech).

### 3.2. Experimental Methods

Freshly synthesized AgN_5_ (127 mg, 0.71 mmol) was introduced into an aqueous solution (15 mL deionized water) containing the chloride salt (0.428 mmol) under continuous stirring. After maintaining the reaction at room temperature for 1 h with agitation, the resulting silver chloride precipitate was separated by filtration. Subsequently, the filtrate was concentrated under reduced pressure to obtain the target compound.

Propamidinium pentazolate (**1**): Yield: 52.196 mg, 85 %. *T*_d_: 118 °C. ^1^H NMR (500 MHz, DMSO-*d*_6_): *δ* = 9.09, 8.79, 2.39, 1.16 ppm. ^13^C NMR (125.72 MHz, DMSO-*d*_6_): *δ* = 172.56, 25.87, 11.51 ppm. IR: *ν* = 3106, 2823, 1702, 1514, 1219, 960 cm^−1^.

5-Amino-4-carbamoyl-1*H*-imidazol-3-ium pentazolate (**2**): Yield: 70.384 mg, 83 %. *T*_d_: 106 °C. ^1^H NMR (500 MHz, DMSO-*d*_6_): *δ* = 14.32, 8.59, 7.41, 6.45 ppm. ^13^C NMR (125.72 MHz, DMSO-*d*_6_): *δ* = 161.58, 143.57, 128.87, 102.95 ppm. IR: *ν* = 3286, 2024, 1581, 1476, 1219, 1065 cm^−1^.

(1*H*-1,2,3-triazol-4-yl)methanaminium pentazolate (**3**): Yield: 63.788 mg, 88 %. *T*_d_: 116 °C. ^1^H NMR (500 MHz, DMSO-*d*_6_): *δ* = 11.95, 8.67, 8.01, 4.12 ppm. ^13^C NMR (125.72 MHz, DMSO-*d*_6_): *δ* = 140.13, 49.01, 34.13 ppm. IR: *ν* = 2932, 2015, 1481, 1383, 1215, 966 cm^−1^.

5-Amino-4*H*-1,2,4-triazol-1-ium pentazolate (**4**): Yield: 54.127 mg, 81 %. *T*_d_: 104 °C. ^1^H NMR (500 MHz, DMSO-*d*_6_): *δ* = 13.68, 8.29, 8.03 ppm. ^13^C NMR (125.72 MHz, DMSO-*d*_6_): *δ* = 151.17, 139.39 ppm. IR: *ν* = 3144, 2047, 1649, 1354, 1229, 944 cm^−1^.

5-Amino-3-methyl-4*H*-1,2,4-triazol-1-ium pentazolate (**5**): Yield: 66.211 mg, 91 %. *T*_d_: 108 °C. ^1^H NMR (500 MHz, DMSO-*d*_6_): *δ* = 7.87, 5.18, 3.60 ppm (s, 2 H). ^13^C NMR (125.72 MHz, DMSO-*d*_6_): *δ* = 151.55, 147.97, 11.37 ppm. IR: *ν* = 2987, 2160, 1689, 1386, 1215, 1058 cm^−1^.

Amino(pyrimidin-2-yl)methaniminium pentazolate (**6**): Yield: 69.770 mg, 81%. *T*_d_: 109 °C. ^1^H NMR (500 MHz, DMSO-*d*_6_): *δ* = 9.54, 9.15, 7.92 ppm. ^13^C NMR (125.72 MHz, DMSO-*d*_6_): *δ* = 160.54, 158.82, 153.33, 125.38 ppm. IR: *ν* = 2987, 2027, 1572, 1399, 1211, 1066 cm^−1^.

### 3.3. Theoretical Methods

The elementary geometric optimization and the frequency analysis were performed at the level of the Becke three parameter, Lee-Yan-Parr (B3LYP) [33] functional with the 6-311++G** basis set. All of the optimized structures were characterized to be local energy minima on the potential surface without any imaginary frequencies. Atomization energies were calculated by the CBS-4M [34].

**Scheme 2:**
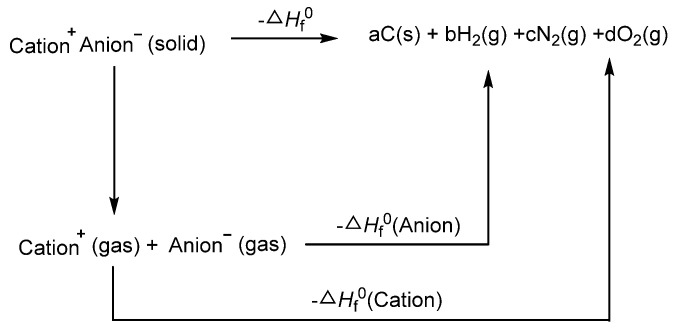
Born-Haber cycle for the formation of energetic salts.

For energetic salts, the solid-phase heats of formation are calculated on the basis of a Born-Haber energy cycle (Scheme 2).

Based on a Born-Haber energy cycle, the heat of formation of a salt can be simplified by the formula given in Equation (1):

Δ*H*_f_ (salt, 298 K) = Δ*H*_f_ (cation, 298 K) + Δ*H*_f_ (anion, 298 K) − Δ*H*_L_ (1)

where Δ*H*_L_ is the lattice energy of the salts, which could be predicted by using the formula suggested by Jenkins et al. [35].

Δ*H*_L_ = *U*_POT_ + [*p*(*n*_M_/2 − 2) + *q*(*n*_X_/2 − 2)]*RT* (2)

where *n*_M_ and *n*_X_ depend on the nature of the ions, M*_p_*^+^ and X*_q_*^-^, and are equal to 3 for monatomic ions, 5 for linear polyatomic ions, and 6 for nonlinear polyatomic ions.

The equation for lattice potential energy *U*_POT_ has the form:

*U*_POT_ [kJ mol^−1^] = *x*(*ρ*/*M*)^1/3^ + *y* (3)

where *ρ*/g cm^−3^ is the density, *M* is the chemical formula mass of the ionic material, and values for the coefficients *x*/kJ mol^−1^ cm and *y*/kJ mol^−1^ are taken from the literature [36].

## 4. Conclusions

In summary, through metathesis reactions between AgN_5_ and corresponding chloride salts, six novel *cyclo*-N_5_^−^ salts were successfully synthesized and comprehensively characterized. Their crystal structures were confirmed through single-crystal X-ray diffraction analyses. Physicochemical and energetic properties were also fully evaluated.

All *cyclo*-N_5_^−^ salts exhibit superior safety characteristics with high mechanical stabilities (IS > 35 J, FS > 360 N), outperforming previous *cyclo*-N_5_^−^ salts N(CH_3_)_4_N_5_, [DAG]N_5_, and [EDA](N_5_)_2_. Thermal analyses in this study demonstrated that **1**–**6** exhibit higher decomposition temperatures (*T*_peak_ ≥ 107 °C) compared to N(CH_3_)_4_N_5_, [DAG]N_5_, and [EDA](N_5_)_2_. These enhanced stabilities can be attributed to their dense hydrogen-bonding networks. Furthermore, all *cyclo*-N_5_^−^ salts demonstrate positive heats of formation in the range of 210.33–533.86 kJ mol^−1^, detonation velocities of 7122–8288 m s^−1^, and detonation pressures of 14.6–25.1 GPa. This work provides valuable insights for crystal engineers to further enhance the stability of *cyclo*-N_5_^−^ salts and explore a broader variety of energetic materials containing *cyclo*-N_5_^−^.

## Figures and Tables

**Table 1:** Physicochemical and energetic properties of compounds **1**–**6**.

Comp.	*ρ* *^a^* (g cm^−3^)	*T*_d_ *^b^* (°C)	IS *^c^* (J)	FS *^d^* (N)	Δ*H*_f_ *^e^* (kJ mol^−1^)	*D* *^f^* (m s^−1^)	*P* *^g^* (GPa)
**1**	1.307	121	>40	>360	213.97	7122	14.6
**2**	1.575	107	>40	>360	210.66	7791	23.3
**3**	1.541	118	>35	>360	533.86	8288	22.6
**4**	1.580	120	>35	>360	440.90	8162	25.1
**5**	1.430	108	>40	>360	402.99	7561	20.0
**6**	1.485	117	>40	>360	376.29	8094	21.0
C_2_H_6_N_10_ [19]	1.583	107	>40	>360	639.7	7824	24.5
NH_4_N_5_ [19]	1.486	106.5	8	130	269.1	7757	23.2
N(CH_3_)_4_N_5_ [14]	1.245	81.6	35	>360	296.1	6300	14.0
[DAG]N_5_ [19]	1.438	100.5	25	240	508.0	7505	21.2
[EDA](N_5_)_2_ [22]	1.395	98.5	25	240	556.1	6896	17.5

*^a^* Density, measured using a gas pycnometer at 25 °C. *^b^* Decomposition temperature (peak). *^c^* Impact sensitivity (IS). *^d^* Friction sensitivity (FS). *^e^* Heat of formation. *^f^* Calculated detonation velocity. *^g^* Calculated detonation pressure. DAG, 1,3-diaminoguanidinium. EDA, monoethane-1,2-diaminium.

## Data Availability

The data presented in this study are available in the paper.

## Supplementary Materials

The following supporting information can be downloaded at: https://www.mdpi.com/article/10.3390/molecules30122613/s1, ^1^H and ^13^C NMR spectra; crystal structure data (PDF).
