# Supplementary material for: Dense Hydrogen-Bonded Assembly of Hydrogen-Rich Cations and Pentazolate Anions: A Series of Highly Insensitive Ionic Salts
Source: Molecules. 2025 Jun 16;30(12):2613. doi: 10.3390/molecules30122613 (PMC12195709; doi:10.3390/molecules30122613)
Supplement: Supplementary file 1 [file molecules-30-02613-s001.zip › molecules-3643653-supplementary.pdf]

Supporting Information

Dense Hydrogen-Bonded Assembly of Hydrogen-Rich Cations and Pentazolate Anions: A Series of Highly Insensitive Ionic Salts

Lianghe Sun, Hongwei Zhu, Shuaijie Jiang, Xiaofeng Yuan, Guoping Lu, Ming Lu * and Yuangang Xu *

School of Chemistry and Chemical Engineering, Nanjing University of Science and Technology, Nanjing 210094, China; 18112254480@163.com (L.S.); 18895545396@163.com (H.Z.); jsj2020@njust.edu.cn (S.J.); yuan_xiaof@163.com (X.Y.); glu@njust.edu.cn (G.L.)

* Correspondence: luming@njust.edu.cn (M.L.); yuangangxu@njust.edu.cn (Y.X.)

Index

1. ^{1}H and ^{13}C NMR spectra

2. Crystal structure data

1. ^{1}H and ^{13}C NMR spectra

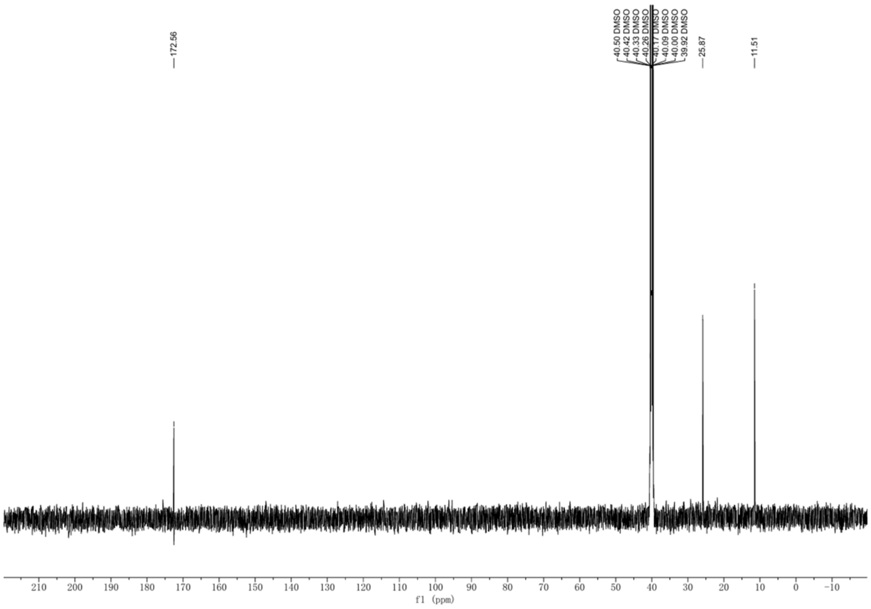

Figure S1. ^{13}C NMR spectrum of **1**.

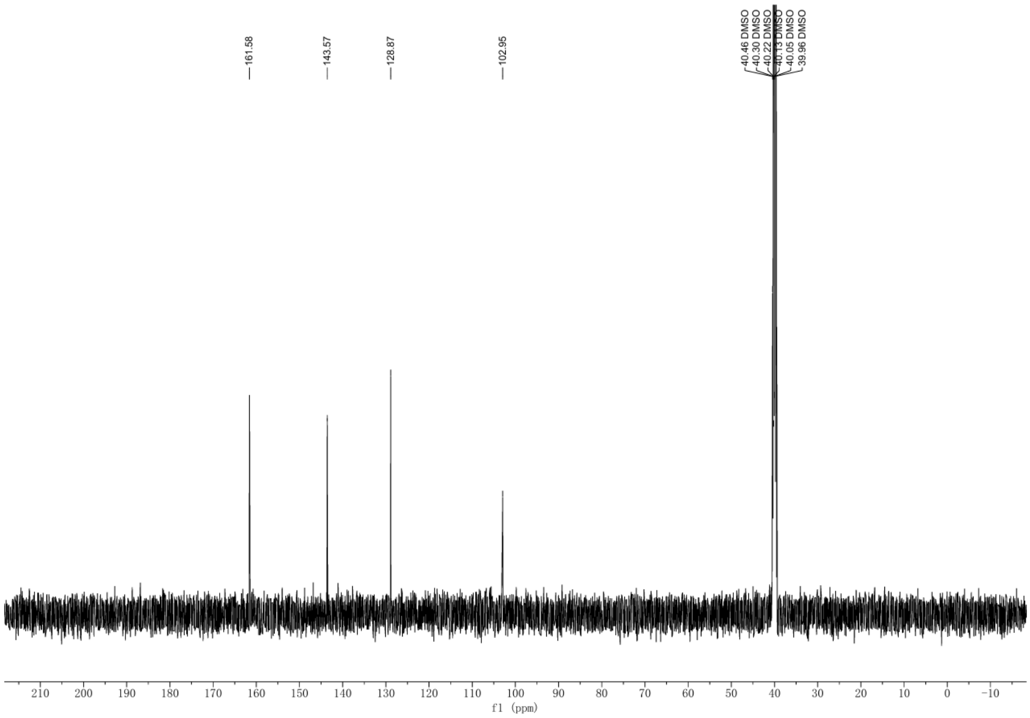

Figure S2. ^{13}C NMR spectrum of **2**.

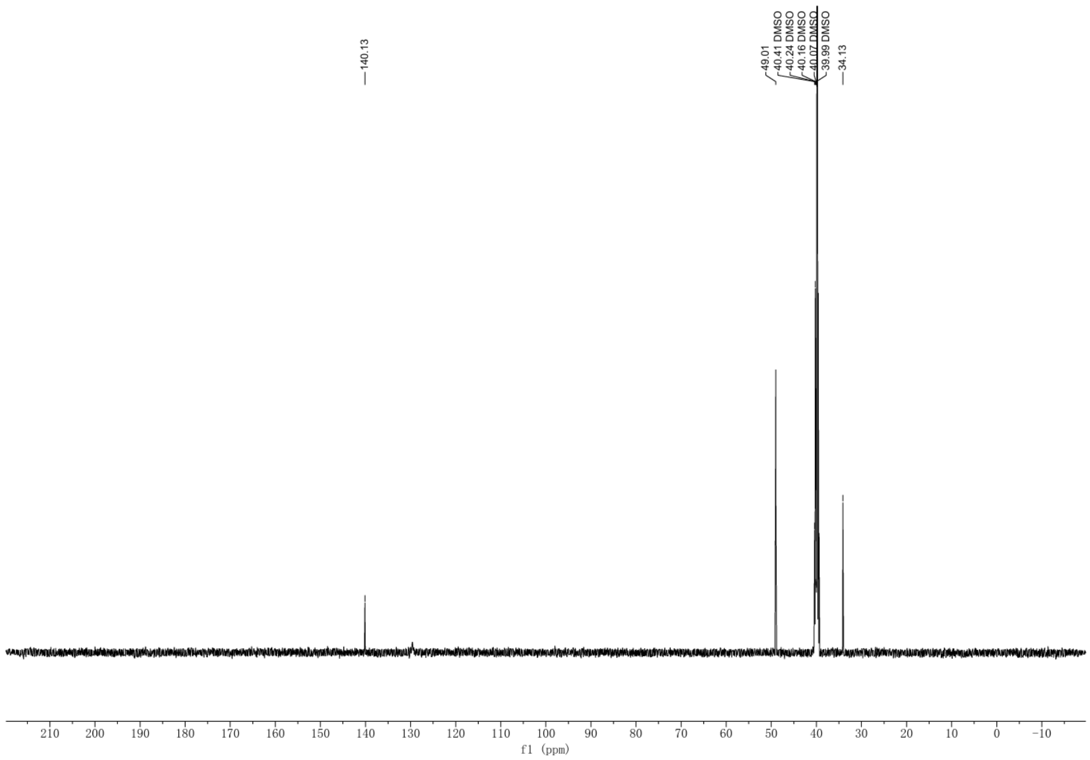

Figure S3. ^{13}C NMR spectrum of **3**.

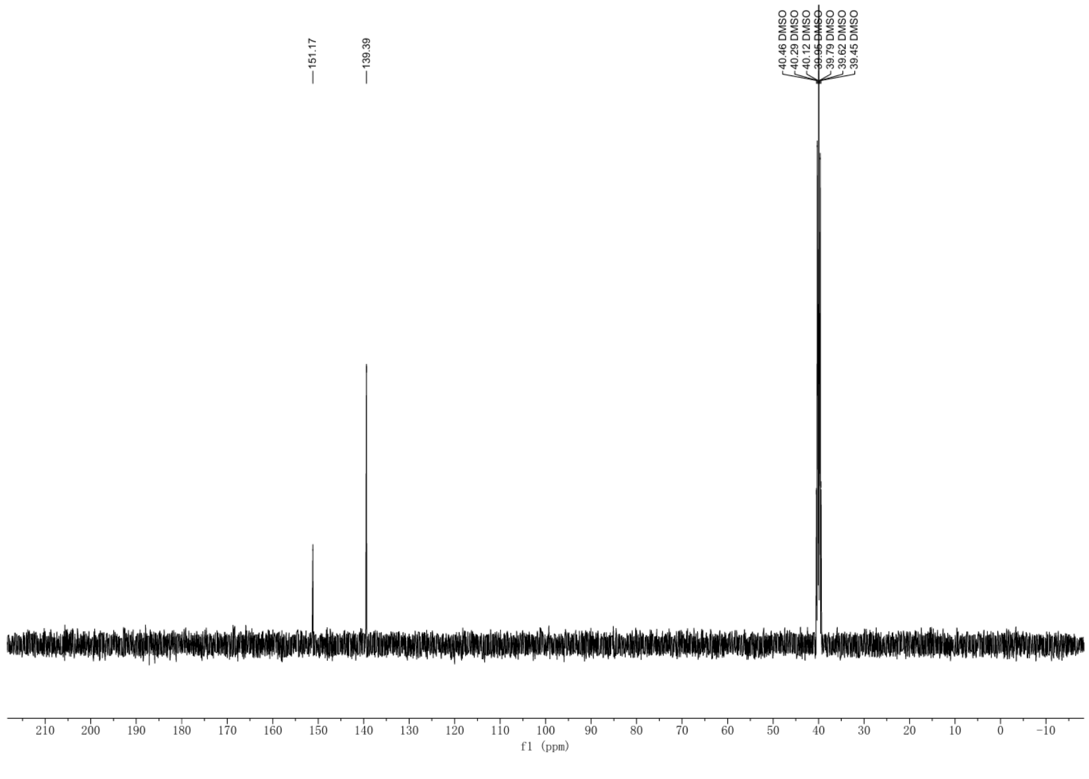

Figure S4. ^{13}C NMR spectrum of **4**.

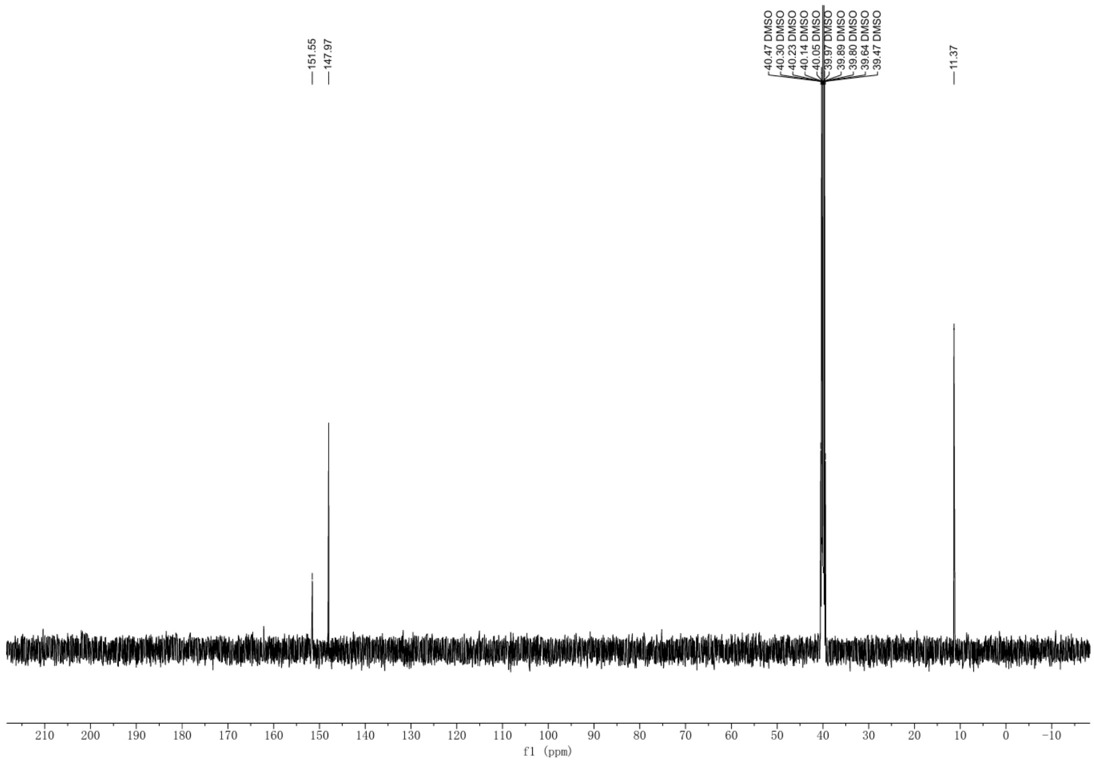

Figure S5. ^{13}C NMR spectrum of **5**.

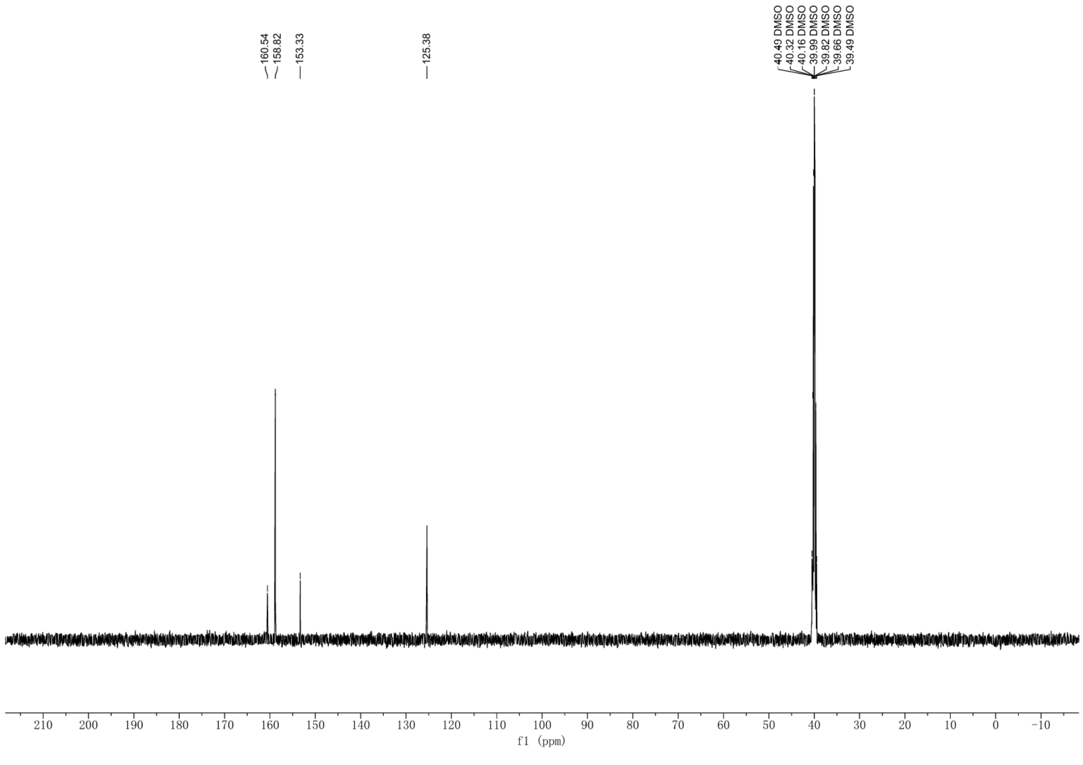

Figure S6. ^{13}C NMR spectrum of **6**.

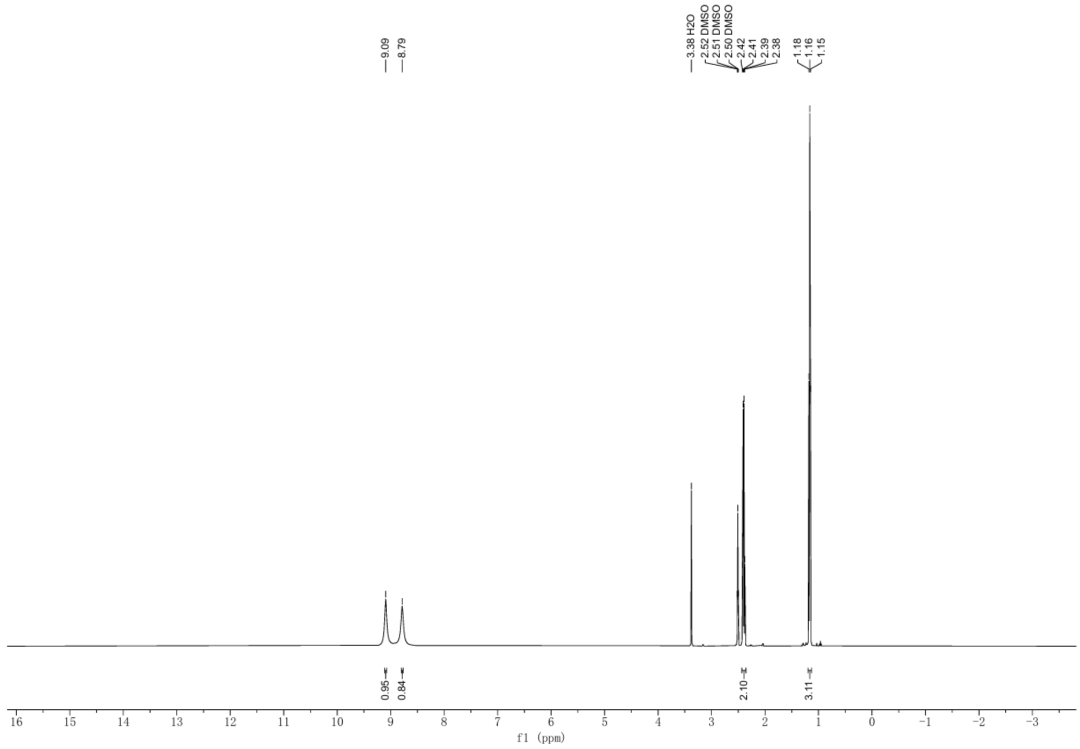

Figure S7. ^{1}H NMR spectrum of **1**.

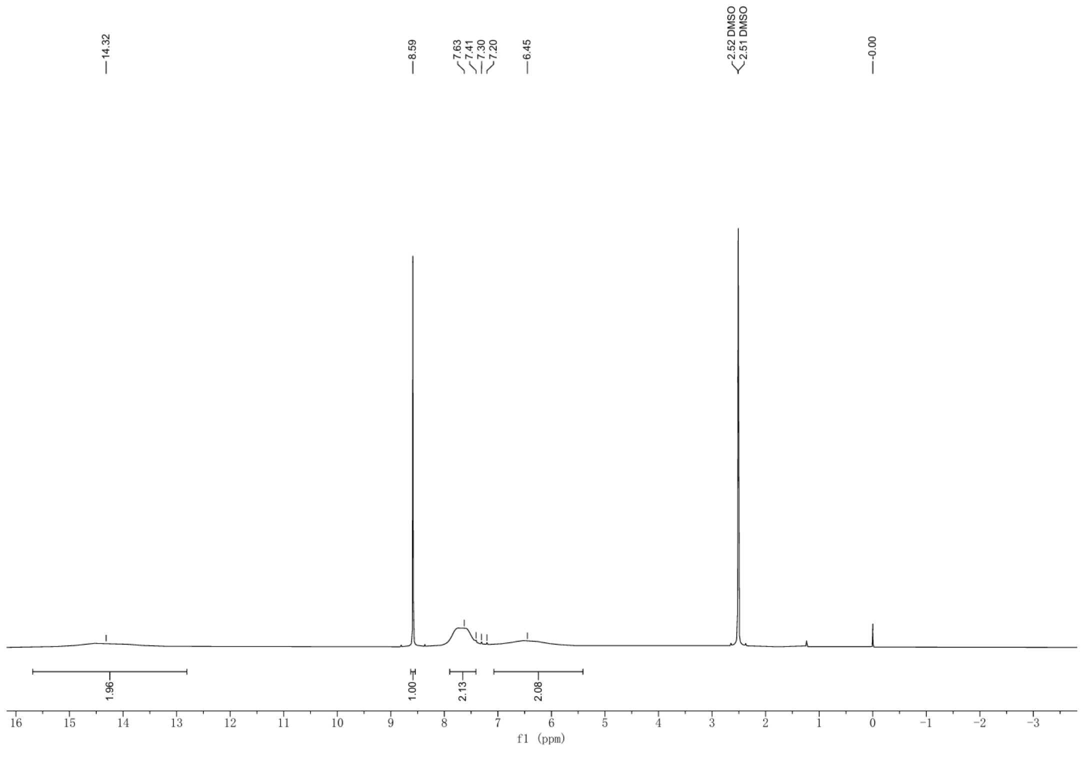

Figure S8. ^{1}H NMR spectrum of **2**.

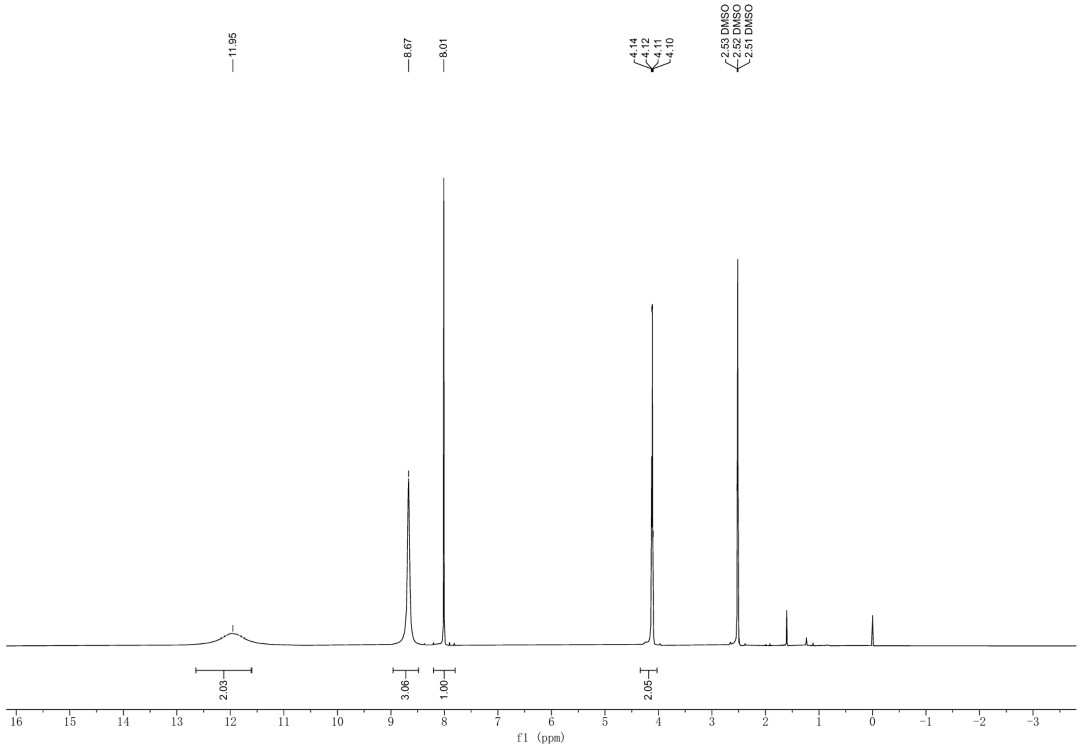

Figure S9. ^{1}H NMR spectrum of **3**.

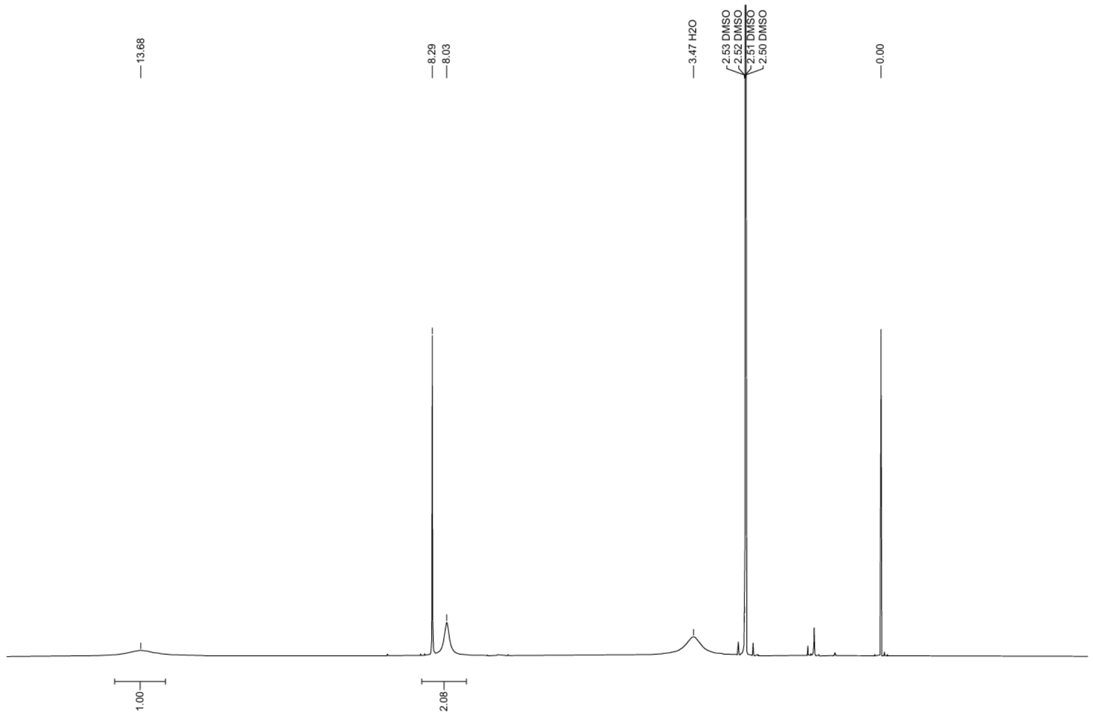

Figure S10. ^{1}H NMR spectrum of **4**

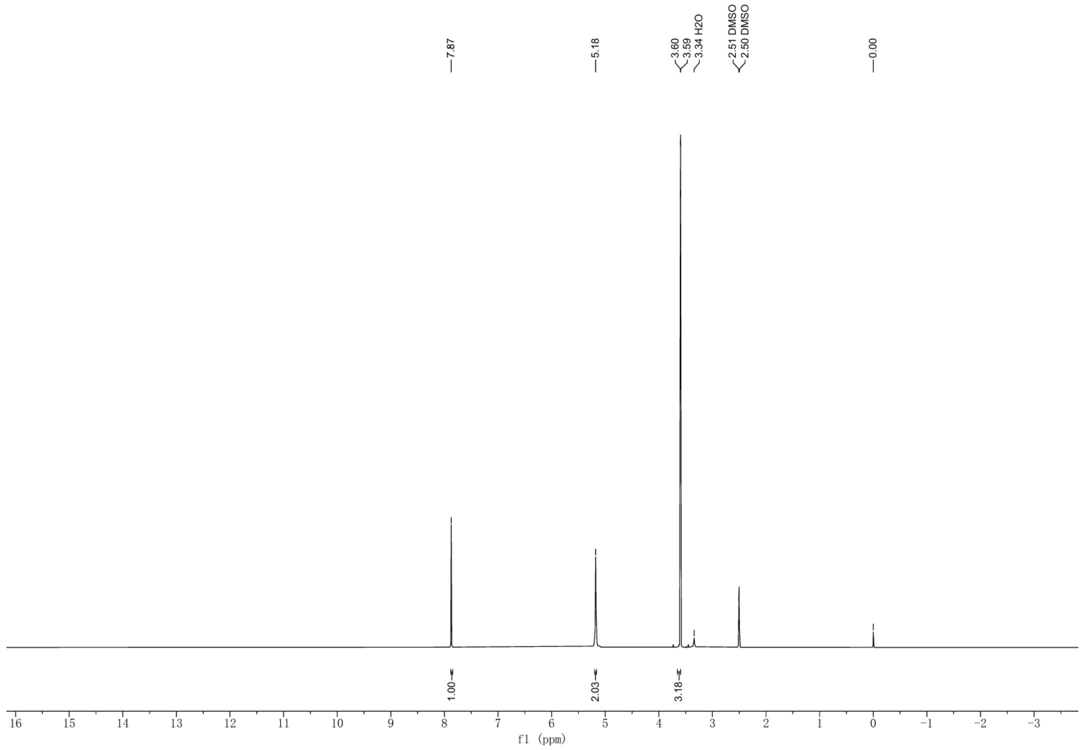

Figure S11. ^{1}H NMR spectrum of **5**.

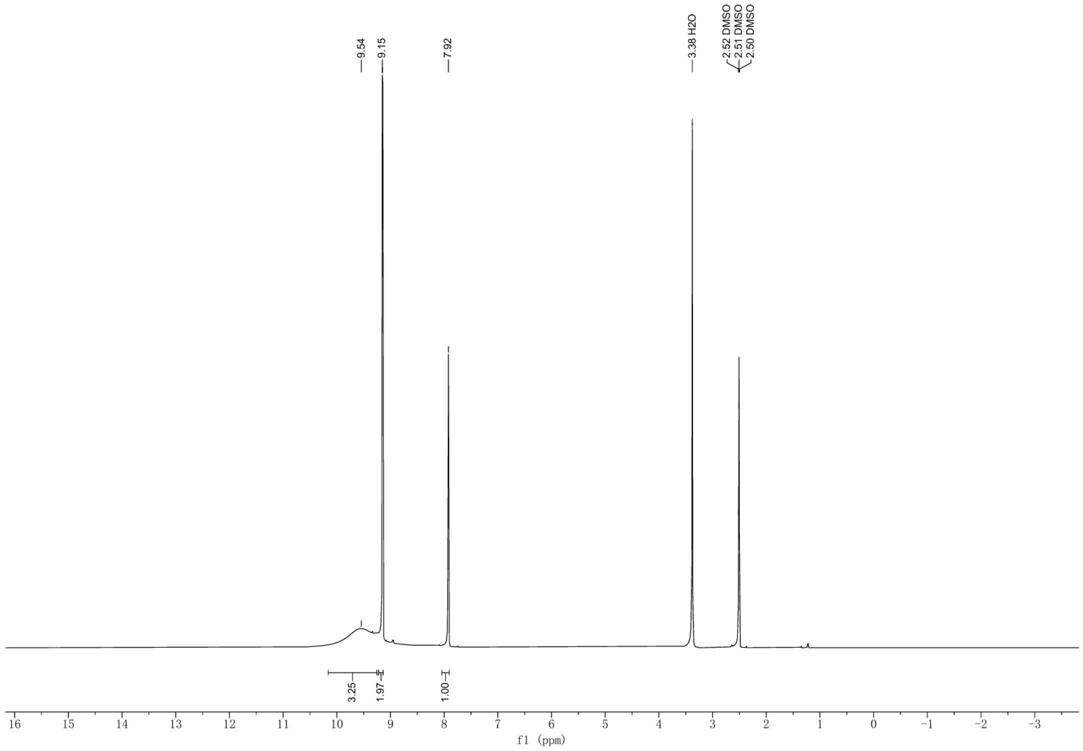

Figure S12. ^{1}H NMR spectrum of **6**.

2. Crystal structure data

Table S1. Crystal data and structure refinement for **1** and **2**·$2H_2O$

Crystal	**1**	**2**·$2H_2O$
CCDC	2446450	2446451
Empirical formula	$C_3H_9N_7$	$C_4H_{11}N_9O_3$
Formula weight	143.17	233.22
Temperature [K]	173K	170K
Crystal system	monoclinic	monoclinic
Space group (number)	$P2_1/m$	$C2/c$
a [Å]	7.1900(10)	23.1334(7)
b [Å]	6.5385(13)	7.6075(3)
c [Å]	7.6739(13)	13.5064(4)
α [Å]	90	90
β [Å]	98.371(8)	122.9990(10)
γ [Å]	90	90
Volume [$Å^3$]	356.92(11)	1993.50(12)
Z	2	8
ρ_{calc} [g cm^{-3}]	1.332	1.554
μ [mm^{-1}]	0.099	0.131
F(000)	152.0	976.0
Crystal size [mm^3]	0.11 × 0.05 × 0.04	0.12 × 0.06 ×0.05
Crystal colour	colourless	colourless
Crystal shape	block	block
Radiation	MoKα (λ=0.71073)	MoKα (λ = 0.71073)
2θ [°]	5.366 to 52.668	4.198 to 52.792
Index ranges	$-7 \leq h \leq 8$<br>$-7 \leq k \leq 8$<br>$-9 \leq l \leq 9$	$-28 \leq h \leq 28$<br>$-9 \leq k \leq 9$,<br>$-16 \leq l \leq 16$
Reflections collected	3156	8956
Data/Restraints/Parameters	792 / 0 / 65	2031 / 10 / 189
Goodness-of-fit on F^2	1.081	1.033
Final R indexes [I >= 2σ (I)]	R1 = 0.0552,<br>wR2 = 0.1340	R1 = 0.0408,<br>wR2 = 0.0928
Final R indexes [all data]	R1 = 0.0735,<br>wR2 = 0.1465	R1 = 0.0581,<br>wR2 = 0.1035
Largest diff. peak / hole / e $Å^{-3}$	0.311 / -0.270	0.22 / -0.18

Table S2. Crystal data and structure refinement for **3** and **4**·$2H_2O$

Crystal	**3**	**4**·$2H_2O$
CCDC	2446452	2446453
Empirical formula	$C_3H_7N_9$	$C_2H_8N_9O_2$
Formula weight	169.18	190.17
Temperature [K]	170	296(2)
Crystal system	monoclinic	monoclinic
Space group (number)	$P2_1/c$	$P2_1/c$
a [Å]	5.0570(2)	10.4710(19)
b [Å]	7.2763(3	13.007(3)
c [Å]	19.5810(7)	6.2137(13)
α [Å]	90	90
β [Å]	96.762(2)	97.214(6)
γ [Å]	90	90
Volume [Å^3]	715.50(5)	839.6(3)
Z	4	4
ρ_{calc} [g cm^{-3}]	1.571	1.504
μ [mm^{-1}]	0.120	0.128
F (000)	352.0	396
Crystal size [mm^3]	0.12 × 0.06 × 0.05	0.180 × 0.160 × 0.150
Crystal colour	colourless	colourless
Crystal shape	block	block
Radiation	MoKα (λ = 0.71073)	MoKα (λ = 0.71073)
2θ [°]	4.19 to 52.698	3.132 to 24.994
Index ranges	-6 ≤ h ≤ 6<br>-9 ≤ k ≤ 8<br>24 ≤ l ≤ 24	-12 <= h <=12<br>15 <= k <= 15<br>-5 <= l <= 7
Reflections collected	5224	1458
Data / Restraints / Paramet ers	1424 / 0 / 125	1458 / 0 / 125
Goodness-of-fit on F^2	1.052	1.091
Final R indexes [I >= 2σ (I)]	R1 = 0.0396,<br>wR2 = 0.0959	R1 = 0.0776,<br>wR2 = 0.2309
Final R indexes [all data]	R1 = 0.0505<br>wR2 = 0.1047	R1 = 0.0909<br>wR2 = 0.2408
Largest diff. peak / hole / e Å^{-3}	0.24 / -0.18	0.421 / -0.509

Table S3. Crystal data and structure refinement for **5**·H_2O and **6**

Crystal	**5**·H_2O	**6**
CCDC	2446454	2446455
Empirical formula	$C_3H_9N_9O$	$C_3H_9N_9$
Formula weight	187.19	171.19

Temperature [K]	150	170
Crystal system	orthorhombic	triclinic
Space group (number)	*Fdd2*	*P-1*
a [Å]	15.7810(6)	6.7888(11)
b [Å]	53.9823(18)	7.8796(11)
c [Å]	3.9929(2)	8.3492(13)
α [Å]	90	62.850(4)
β [Å]	90	70.961(6)
γ [Å]	90	80.786(6)
Volume [$Å^3$]	3401.5(2)	375.64(10)
Z	16	2
ρ_{calc} [g cm^{-3}]	1.462	1.514
μ [mm^{-1}]	1.005	0.115
F (000)	1568.0	180.0
Crystal size [mm^3]	0.11 × 0.04 × 0.02	0.12 × 0.06 × 0.04
Crystal colour	colourless	colourless
Crystal shape	block	block
Radiation	CuKα (λ = 1.54178)	MoKα (λ = 0.71073)
2θ [°]	6.55 to 127.358	5.726 to 52.73
Index ranges	-18 ≤ h ≤ 17<br>-62 ≤ k ≤ 57<br>-4 ≤ l ≤ 4	-8 ≤ h ≤ 8<br>-9 ≤ k ≤ 9<br>-10 ≤ l ≤ 10
Reflections collected	6617	4141
Data / Restraints / Parameters	1397 / 1 / 144	1526 / 0 / 129
Goodness-of-fit on F^2	1.044	1.079
Final R indexes [I >= 2σ (I)]	R1 = 0.0476,<br>wR2 = 0.1217	R1 = 0.0624,<br>wR2 = 0.1329
Final R indexes [all data]	R1 = 0.0525,<br>wR2 = 0.1279	R1 = 0.1348,<br>wR2 = 0.1652
Largest diff. peak / hole / e $Å^{-3}$	0.21 / -0.23	0.26 / -0.28

Table S4. Bond lengths for **1**

parameter	bond length (Å)	parameter	bond length (Å)
N6-N7	1.311(3)	N4-N5	1.316(3)
N6-N5	1.319(3)	N4-N3	1.320(3)
N2-C1	1.297(4)	C1-C2	1.498(4)
N1-C1	1.313(3)	C2-C3	1.370(5)
N7-C3	1.320(3)		

Table S5. Bond angles for **1**

parameter	bond angle (°)	parameter	bond angle (°)
N7-N6-N5	108.1(2)	N2-C1-N1	121.7(2)
N6-N7-N3	108.2(2)	N2-C1-C2	118.2(3)
N5-N4-N3	108.1(2)	N1-C1-C2	120.0(3)
N4-N5-N6	107.9(2)	C3-C2-C1	120.8(3)
N7-N3-N4	107.6(2)		

Table S6. Hydrogen bonds for **1**

Donor--H...Acceptor	D-H (Å)	H...A (Å)	D...A (Å)	D-H...A (°)
N1-H1A...N3	0.88	2.20	3.066(3)	170
N1-H1B...N6	0.88	2.13	2.994(3)	166
N2-H2A...N7	0.88	2.15	2.997(3)	162
N2-H2B...N4	0.88	2.14	3.021(3)	174
C3-H3B...N5	0.98	2.55	3.523(5)	174
C3-H3C...N6	0.98	2.61	3.523(5)	156

Table S7. Torsion angles for **1**

parameter	Torsion angle (°)	parameter	Torsion angle (°)
N6-N7-N3-N4	0.000(1)	N5-N6-N7-N3	0.000(1)
N2-C1-C2-C3	149.6(4)	N5-N4-N3-N7	0.000(1)
N1-C1-C2-C3	-30.4(4)	N3-N4-N5-N6	0.000(1)
N7-N6-N5-N4	0.000(1)		

Table S8. Bond lengths for **2**·$2H_2O$

parameter	bond length (Å)	parameter	bond length (Å)
O4-C3	1.253(2)	N6-C3	1.324(2)
N4-C1	1.399(2)	N11-N7	1.321(2)
N4-C4	1.309(2)	N11-N10	1.319(2)
N3-C2	1.375(2)	N7-N8	1.316(2)
N3-C4	1.335(2)	N5-C2	1.350(2)
N9-N10	1.320(2)	C1-C3	1.455(2)
N9-N8	1.314(2)	C2-C2	1.374(2)

Table S9. Bond angles for **2**·$2H_2O$

parameter	bond angle (°)	parameter	bond angle (°)
C4-N4-C1	109.14(14)	C2-C1-C3	126.50(15)

C4-N3-C2	109.04(15)	O4-C3-N6	122.67(16)
N8-N9-N10	108.13(14)	O4-C3-C1	116.98(15)
N10-N11-N7	107.50(14)	N6-C3-C1	120.34(15)
N8-N7-N11	108.39(14)	N5-C2-N3	122.31(16)
N11-N10-N9	108.09(14)	N5-C2-C1	130.93(16)
N9-N8-N7	107.88(14)	C1-C2-N3	106.73(14)
N4-C1-C3	127.57(15)	N4-C4-N3	109.17(15)
C2-C1-N4	105.93(14)		

Table S10. Hydrogen bonds for **2**·2H_2O

Donor--H...Acceptor	D-H (Å)	H...A (Å)	D...A (Å)	D-H...A (°)
N3-H3...O3	0.88(2)	1.91(2)	2.783(2)	170(2)
O3-H3A...N10	0.86(3)	2.05(2)	2.882(2)	162(2)
O3-H3B...O4	0.88(2)	1.92(2)	2.776(2)	165(2)
N4-H4...O5	0.876(18)	1.860(19)	2.721(2)	168(2)
N5-H5A...O4	0.89(2)	2.28(2)	2.864(4)	123.6(16)
N5-H5A...N8	0.89(2)	2.29(2)	2.994(2)	137.0(17)
N5-H5B...O4	0.869(19)	2.097(18)	2.956(2)	170(3)
O5-H5C...N7	0.87(2)	1.99(2)	2.856(2)	175(3)
O5-H5D...N11	0.87(2)	2.03(2)	2.883(2)	166(2)
N6-H6A...N9	0.88(2)	2.22(2)	3.099(3)	175(2)
N6-H6B...O5	0.879(19)	2.22(2)	3.078(2)	166(2)
C4-H4A...O3	0.93(2)	2.37(2)	3.246(5)	157(19)

Table S11. Torsion angles for **2**·2H_2O

parameter	Torsion angle (°)	parameter	Torsion angle (°)
N4-C1-C3-O4	-177.68(16)	C3-C1-C2-N3	-179.92(16)
N4-C1-C3-N6	2.60(3)	C3-C1-C2-N5	2.2(3)
N4-C1-C2-N3	-0.48(18)	C2-N3-C4-N4	-0.7(2)
N4-C1-C2-N5	-178.39(18)	C2-C1-C3-O4	1.6(3)
N11-N7-N8-N9	0.30(2)	C2-C1-C3-N6	-178.08(17)
N7-N11-N10-N9	0.10(2)	C4-N4-C1-C3	179.48(17)
N10-N9-N8-N7	-0.20(2)	C4-N4-C1-C2	0.05(19)
N10-N11-N7-N8	-0.20(2)	C4-N3-C2-N5	178.89(17)
N8-N9-N10-N11	0.00(2)	C4-N3-C2-C1	0.75(19)
C1-N4-C4-N3	0.40(2)		

Table S12. Bond lengths for **3**

parameter	bond length (Å)	parameter	bond length (Å)
N9-C3	1.493(2)	N1-N5	1.318(2)

N6-N7	1.3252(17)	N3-N2	1.3156(19)
N6-N8	1.3239(18)	N3-N4	1.316(2)
N7-C1	1.3443(19)	N5-N4	1.313(2)
N8-C2	1.3368(19)	C1-C3	1.4924(19)
N1-N2	1.3107(18)	C1-C2	1.386(2)

Table S13. Bond angles for **3**

parameter	bond angle (°)	parameter	bond angle (°)
N8-N6-N7	116.13(12)	N5-N4-N3	107.99(13)
N6-N7-C1	103.29(12)	N7-C1-C3	122.74(13)
N6-N8-C2	103.07(12)	N7-C1-C2	108.25(13)
N2-N1-N5	108.26(12)	C2-C1-C3	129.00(14)
N2-N3-N4	108.10(12)	C1-C3-N9	112.58(12)
N1-N2-N3	107.85(13)	N8-C2-C1	109.26(14)
N4-N5-N1	107.80(13)		

Table S14. Hydrogen bonds for **3**

Donor--H...Acceptor	D-H (Å)	H...A (Å)	D...A (Å)	D-H...A (°)
N6-H6...N1	0.89(2)	2.07(2)	2.8565(19)	146.7(19)
N9-H9A...N7	0.85(2)	2.27(2)	3.0797(18)	159(2)
N9-H9B...N2	0.93(2)	2.02(2)	2.900(2)	158.2(17)
N9-H9C...N3	0.96(2)	1.96(19)	2.9130(19)	175.9(19)
C2-H2...N5	0.95	2.61	3.393(2)	140
C3-H3A...N4	0.99	2.46	3.415(2)	162

Table S15. Torsion angles for **3**

parameter	Torsion angle (°)	parameter	Torsion angle (°)
N6-N7-C1-C3	178.84(13)	N1-N5-N4-N3	-0.06(18)
N6-N7-C1-C2	0.09(16)	N2-N1-N5-N4	0.20(18)
N6-N8-C2-C1	0.34(17)	N2-N3-N4-N5	-0.10(18)
N7-N6-N8-C2	-0.30(18)	N5-N1-N2-N3	-0.26(18)
N7-C1-C3-N9	83.74(18)	N4-N3-N2-N1	0.23(18)
N7-C1-C2-N8	-0.28(18)	C3-C1-C2-N8	-178.92(14)
N8-N6-N7-C1	0.13(17)	C2-C1-C3-N9	-97.80(19)

Table S16. Bond lengths for **4**·$2H_2O$

parameter	bond length (Å)	parameter	bond length (Å)
C1-N6	1.322(4)	N1-N5	1.311(4)

C1-N9	1.329(5)	N2-N3	1.311(4)
C1-N8	1.337(4)	N3-N4	1.316(4)
C2-N7	1.292(5)	N4-N5	1.307(4)
C2-N8	1.359(5)	N6-N7	1.385(4)
N1-N2	1.308(5)		

Table S17. Bond angles for **4**·$2H_2O$

parameter	bond angle (°)	parameter	bond angle (°)
N6-C1-N9	126.9(3)	N2-N3-N4	107.5(3)
N6-C1-N8	106.3(3)	N5-N4-N3	108.2(3)
N9-C1-N8	126.8(3)	N4-N5-N1	107.9(3)
N7-C2-N8	111.9(3)	C1-N6-N7	111.1(3)
N2-N1-N5	108.1(3)	C2-N7-N6	103.7(3)
N1-N2-N3	108.2(3)	C1-N8-C2	107.0(3)

Table S18. Hydrogen bonds for **4**·$2H_2O$

Donor--H...Acceptor	D-H (Å)	H...A (Å)	D...A (Å)	D-H...A (°)
C2-H2...N2	0.93	2.69	3.440(5)	137.7
C2-H2...O1	0.93	2.56	3.258(5)	131.8
N6-H6...O2	0.86	1.94	2.693(4)	146.1
N8-H8...O1	0.86	1.86	2.715(4)	174.6
N9-H9A...N4	0.86	2.15	2.961(4)	156.5
N9-H9B...N7	0.86	2.22	3.059(4)	165.0
O1-H1A...N5	0.84(19)	2.05(2)	2.872(4)	168(4)
O1-H1B...N2	0.81(4)	2.07(4)	2.931(4)	175(4)
O2-H2A...N3	0.78	2.16	2.883(4)	155.1
O2-H2B...N1	1.03	1.98	2.956(4)	156.8

Table S19. Torsion angles for **4**·$2H_2O$

parameter	Torsion angle (°)	parameter	Torsion angle (°)
C1-N6-N7-C2	0.5(3)	N6-C1-N8-C2	0.4(3)
N1-N2-N3-N4	-0.5(4)	N7-C2-N8-C1	-0.1(4)
N2-N1-N5-N4	0.5(4)	N8-C1-N6-N7	-0.5(3)
N2-N3-N4-N5	0.9(4)	N8-C2-N7-N6	-0.2(4)
N3-N4-N5-N1	-0.8(4)	N9-C1-N6-N7	178.5(3)
N5-N1-N2-N3	0.0(4)	N9-C1-N8-C2	-178.7(3)

Table S20. Bond lengths for **5**·H_2O

parameter	bond length (Å)	parameter	bond length (Å)
N8-C1	1.350(5)	C2-C3	1.487(5)
N8-C2	1.378(5)	N1-N2	1.317(5)
N7-N6	1.391(4)	N1-N5	1.317(5)
N7-C2	1.296(5)	N2-N3	1.315(4)
N6-C1	1.327(5)	N3-N4	1.319(5)
N9-C1	1.314(5)	N5-N4	1.321(5)

Table S21. Bond angles for **5**·H_2O

parameter	bond angle (°)	parameter	bond angle (°)
C1-N8-C2	107.3(3)	N7-C2-N8	111.3 (3)
C2-N7-N6	104.0(3)	N7-C2-C3	126.1(4)
C1-N6-N7	111.8(3)	N2-N1-N5	108.8(3)
N6-C1-N8	105.7(3)	N3-N2-N1	107.8(3)
N9-C1-N8	126.2(4)	N2-N3-N4	107.8(3)
N9-C1-N6	128.1(4)	N1-N5-N4	107.1(3)
N8-C2-C3	122.7(4)	N3-N5-N4	108.5(3)

Table S22. Hydrogen bonds for **5**·H_2O

Donor--H...Acceptor	D-H (Å)	H...A (Å)	D...A (Å)	D-H...A (°)
O1-H1A...N2	0.87(6)	2.01(5)	2.852(5)	163(5)
O1-H1B...N3	0.93(7)	1.93(7)	2.845(5)	166(5)
N6-H6...N1	0.92(5)	2.03(5)	2.944(5)	176(3)
N8-H8...N4	0.92(5)	1.98(5)	2.903(4)	179(5)
N9-H9A...O1	0.88(5)	1.96(5)	2.824(4)	169(4)
N9-H9B...O1	0.85(5)	2.01(5)	2.846(5)	168(4)

Table S23. Torsion angles for **5**·H_2O

parameter	Torsion angle (°)	parameter	Torsion angle (°)
N7-N6-C1-C8	0.3(5)	C2-N8-C1-N9	179.7(4)
N7-N6-C1-N9	-179.5(4)	C2-N7-N6-C1	-0.3(5)
N6-N7-C2-N8	0.3(5)	N1-N2-N3-N4	0.1(4)
N6-N7-C2-C3	-179.4(4)	N1-N5-N4-N3	-0.2(5)
C1-N8-C2-N7	-0.2(5)	N2-N1-N5-N4	0.3(4)
C1-N8-C2-C3	179.6(4)	N2-N3-N4-N5	0.1(4)
C2-N8-C1-N6	-0.1(5)	N5-N1-N2-N3	-0.2(4)

Table S24. Bond lengths for **6**

parameter	bond length (Å)	parameter	bond length (Å)
N7-C3	1.323(3)	N3-N2	1.321(3)
N7-C2	1.466(3)	N3-N4	1.308(4)
N6-C3	1.327(4)	N2-N1	1.309(4)
N6-C1	1.467(4)	N4-N5	1.320(4)
N8-N9	1.403(4)	N1-N5	1.315(3)
N8-C3	1.312(4)	C1-C2	1.526(4)

Table S25. Bond angles for **6**

parameter	bond angle (°)	parameter	bond angle (°)
C3-N7-C2	110.2(2)	N7-C3-N6	111.0(3)
C3-N6-C1	110.0(2)	N8-C3-N7	124.4(3)
C3-N8-N9	117.6(2)	N8-C3-N6	124.6(2)
N4-N3-N2	107.7(2)	N1-N5-N4	107.5(3)
N1-N2-N3	108.1(2)	N6-C1-C2	101.3(2)
N3-N4-N5	108.4(2)	N7-C2-C1	101.5(2)
N2-N1-N5	108.3(2)		

Table S26. Hydrogen bonds for **6**

Donor--H...Acceptor	D-H (Å)	H...A (Å)	D...A (Å)	D-H...A (°)
N11-H11A...N5	0.88	2.59	2.927(4)	104
N11-H11A...N1	0.88	2.08	2.923(4)	160
N11-H11B...N14	0.88	2.33	2.692(4)	105
N12-H12A...N1	0.88	2.62	3.322(4)	137
N12-H12A...N2	0.88	2.16	3.022(4)	167
N12-H12B...N10	0.88	2.29	3.070(4)	148
N12-H12B...N13	0.88	2.36	2.707(4)	104
N17-H17A...N7	0.88	2.07	2.926(4)	165
N17-H17B...N16	0.88	2.31	3.046(4)	142
N18-H18A...N8	0.88	2.06	2.917(4)	164
N18-H18B...N9	0.88	2.11	2.910(4)	151
N18-H18B...N15	0.88	2.29	2.652(4)	105
C4-H4...N4	0.95	2.52	3.345(4)	145
C5-H5...N6	0.95	2.59	3.363(5)	139

Table S27. Torsion angles for **6**

parameter	Torsion angle (°)	parameter	Torsion angle (°)
N6-C1-C2-N7	23.1(3)	N4-N3-N2-N1	0.9(4)

N9-N8-C3-N7	4.0(5)	C3-N7-C2-C1	-19.7(3)
N9-N8-C3-N6	-177.0(3)	C3-N6-C1-C2	-21.0(3)
N3-N2-N1-N5	-0.7(4)	C1-N6-C3-N7	9.6(4)
N3-N4-N5-N1	0.3(4)	C1-N6-C3-N8	-169.6(3)
N2-N3-N4-N5	-0.7(4)	C2-N7-C3-N6	7.2(4)
N2-N1-N5-N4	0.2(4)	C2-N7-C3-N8	-173.6(3)
